# Clinical practice variability: a systematic review of shock wave therapy for spasticity

**DOI:** 10.1093/eurpub/ckac131.301

**Published:** 2022-10-25

**Authors:** R Faubel, IM Martínez, O Navarro, N Sempere-Rubio

**Affiliations:** 1 Department of Physiotherapy, Universitat de València, Valencia, Spain; 2 Asociación de Familiares y Enfermos Parkison, Centro de Rehabilitación Neurológica, Villarobledo, Spain; 3 Research Unit in Clinical Biomechanics, Universitat de València, Valencia, Spain; 4 Department of Nursing, Catholic University of Valencia, Valencia, Spain; 5 Instituto ITACA, Universitat Politècnica de València, Valencia, Spain; 6 Joint Research Unit in Biomedical Engineering, IIS La Fe-Universitat Politècnica de València, Valencia, Spain; 7 PTinMOTION Research Group, Universitat de València, València, Spain

## Abstract

**Background:**

The purpose of this study was to collect and analyse the available scientific evidence on the clinical practice variability and effectiveness of shock wave therapy as a treatment for spasticity.

**Methods:**

the systematic search was performed in the following databases: PubMed, PEDro, Cochrane, Embase, and the Virtual Health Library. All publications from November 2009 to November 2019 were selected that included a sample of patients with spasticity and prior suspension of botulinum toxin, to whom shock wave therapy was applied. The methodological quality of the articles was evaluated using the Jadad scale and the pyramid of quality of scientific evidence.

**Results:**

25 studies involving 866 participants with spasticity were selected. The results obtained suggest that shock wave therapy appears to be effective in reducing spasticity levels irrespective of the age of the participants, the type of injury, and the tool used to measure the effect.

**Conclusions:**

shock wave therapy reports evidence of improvement in motor function, motor impairment, pain, and functional independence, applied independently of botulinum toxin. However, due to the heterogeneity of the protocols, there is no optimum protocol for its application, and it would be appropriate to gain more high-quality scientific evidence through primary studies.

**Key messages:**

• Shock wave therapy reports evidence of improvement in motor function, motor impairment, pain, and functional independence, applied independently of botulinum toxin.

• Due to the heterogeneity of the protocols, there is no optimum protocol for its application, and it would be appropriate to gain more high-quality scientific evidence through primary studies.

